# Risk factors for non-benefit of implantable cardioverter defibrillator therapy

**DOI:** 10.1038/s41598-025-86022-x

**Published:** 2025-01-20

**Authors:** Fabienne Kreimer, Marie Lewenhardt, Ibrahim El-Battrawy, Arash Haghikia, Michael Gotzmann

**Affiliations:** 1 Department of Cardiology II – Rhythmology, University Hospital Münster, Münster, Germany; 2 Department of Cardiology and Rhythmology, St. Josef-Hospital of the Ruhr University Bochum, Gudrunstraße 56, 44791 Bochum, Germany; 3 Institute of Physiology, Department of Cellular and Translational Physiology, Medical Faculty, Ruhr University Bochum, Bochum, Germany; 4 Institut für Forschung und Lehre (IFL), Molecular and Experimental Cardiology, Ruhr University Bochum, Bochum, Germany; 5 Department of Cardiology, Angiology and Intensive care medicine, Deutsches Herzzentrum der Charité, Berlin, Germany; 6 German Center for Cardiovascular Research (DZHK), Partner Site Berlin, Berlin, Germany; 7 Friede Springer Cardiovascular Prevention Center at Charité, Berlin, Germany

**Keywords:** Implantable cardioverter defibrillator, Benefit, Non-benefit, Risk factors, Comorbidities, Age, Cardiology, Cardiac device therapy, Risk factors

## Abstract

Studies have demonstrated overall prognostic benefits of ICD implantation in patients at increased risk of sudden cardiac death. However, results are inconsistent in certain subgroups. This study aims to evaluate the prognostic implications of comorbidities on ICD outcomes and compare trends in patient selection and outcomes over a decade-long inclusion period. This study analysed 422 patients undergoing ICD implantation between 2011 and 2020. The study endpoint “no-benefit” was characterized by death from any cause occurring without prior appropriate ICD therapy. Benefit of ICD implantation was defined as either receiving appropriate ICD therapy before death or surviving until the end of the observation period. During a mean follow-up of 4.2 ± 3.0 years, no-benefit of ICD implantation was observed in 84 patients (20%). Independent risk factors for no-benefit were age ≥ 68 years (HR 4.599, *p* < 0.001), anemia (HR 2.549, *p* < 0.001), peripheral artery disease (HR 2.066, *p* = 0.007), and chronic obstructive pulmonary disease (HR 1.939, *p* = 0.014). Subgroup analysis by age < 68 years and ≥ 68 years demonstrated that the risk of no-benefit increases with age and comorbidities. When comparing patients with ICD implantation in 2011–2015 with those in 2016–2020, there were no significant differences in one-, two- and three-year-no-benefit rates. Different comorbidities were associated with no-benefit in the early and late implantation groups. Risk factors such as older age and specific comorbidities are associated with a higher likelihood of no-benefit from ICD implantation. A careful patient selection and consideration of individual risk factors besides advanced age is important.

**Supplementary Information:**

The online version contains supplementary material available at 10.1038/s41598-025-86022-x.

## Introduction

For over two decades, the implantation of an implantable cardioverter defibrillator (ICD) has been a standard therapy for the prevention of sudden cardiac death, both in primary and secondary prevention^1–5^. This procedure has become routine and around 100,000 patients are undergoing ICD treatment each year in the European Union^6^.

While randomized-controlled studies have demonstrated overall prognostic benefits^1–5^, results are inconsistent in certain subgroups, including patients with end-stage renal disease, non-ischemic cardiomyopathy, diabetes mellitus, and especially the elderly^5–11^. A meta-analysis suggests that patients with a higher comorbidity burden may derive less benefit from primary prevention ICD therapy compared to those with fewer comorbidities^12^. Therefore, the question of which patients benefit from ICD implantation remains critical and procedural as well as short and long-term risks must also be considered.

Ethical concerns complicate the conduct of a randomized trial to assess the improvement in prognosis with an ICD, as guidelines strongly recommend ICD implantation^13^. However, a benefit can be inferred if appropriate ICD therapy successfully treats ventricular tachycardia or ventricular fibrillation. Conversely, the lack of benefit can be concluded if patients die without receiving appropriate ICD therapy.

The aim of this study was to evaluate the prognostic implications of distinct comorbidities on the outcome of patients with ICD and to identify risk factors for non-benefit of ICD therapy. Since the present study covers a long inclusion period over an entire decade, a further aim is to compare the first half of the decade with the second half of the decade to investigate possible trends in patient selection and outcome of ICD patients. In a previous analysis, we were able to highlight the predictive value of age for the benefit and non-benefit of ICD implantation. In this study, in addition to age, we aimed to examine the impact of comorbidities and risk factors in more detail^14^.

## Methods

This retrospective study investigated all patients who underwent ICD implantation at university hospital St Josef Hospital Bochum between 2011 and 2020. This study is an analysis of our previous study, which analysed the benefit of ICD implantation in different age groups^14^. In the present study, the focus is on the analysis of comorbidities and the impact on non-benefit of ICD implantation. Patients lacking follow-up ICD interrogation after hospital discharge were excluded. The analysis encompassed various types of ICD therapy: endovascular and subcutaneous, with or without cardiac resynchronization therapy (CRT), and involved both initial implantations and generator replacements. The indications for ICD implantation were in accordance with the guidelines applicable at the time. Patients gave informed consent. This study was performed in line with the principles of the Declaration of Helsinki. Approval was received from the local ethics committee of Ruhr University Bochum (Number 21–7439-BR).

The primary study endpoint was “no benefit” defined as death from any cause without prior appropriate ICD therapy. Conversely, “benefit of ICD implantation” was defined as receiving appropriate ICD therapy either before death or surviving until the end of the observation period. Appropriate ICD therapy was present in case of antitachycardia pacing [ATP] and/or ICD shock due to ventricular fibrillation or sustained ventricular tachycardia. The third group consisting of surviving patients without prior appropriate ICD therapy was considered to have a neutral outcome.

Patient data, including medical history, medication, laboratory results, ECG, and echocardiography at the time of ICD implantation, were collected. Anemia was defined as a hemoglobin level < 13 g/dL in men and < 12 g/dL in women. The estimated glomerular filtration rate (eGFR) was calculated using the MDRD formula. Kidney disease was defined as eGFR < 60 mL/min. All patients underwent routine check-ups and device interrogations at our outpatient clinic six weeks after implantation, followed by regular checks every six months either at our university outpatient clinic or at a cardiologist’s practice. Interrogations of the patients’ ICDs were evaluated for the assessment of ICD shocks and ATP. The study’s analysis focused on the first appropriate ICD therapy and the corresponding arrhythmia, as well as inappropriate ICD therapy and other device-related complications. Survival analysis was conducted between November 2021 and April 2022, utilizing data from routine examinations at our university outpatient clinic and via telephone contact with patients or their primary care physicians in cases of deceased patients.

The statistical software SPSS 29 (version 29.0.2.0, IBM) was used to conduct the statistical analysis. The numerical data are given as mean ± standard deviation. For the comparison of continuous variables between groups, an unpaired t-test was used for normally distributed variables, while the Mann-Whitney U test or Kruskal-Wallis test was used for non-normally distributed variables. Categorical variables were compared using either the χ2 analysis or Fisher’s exact test. All variables in Table 1 were analyzed for the study endpoint “no benefit of ICD implantation” in a univariate Cox proportional hazard model. All variables with a significant association (illustrated in Table 2) were entered into a multivariate Cox model to identify independent predictors of “no benefit”. Receiver operating characteristic curves were generated to define cut-off values for independent predictors for distinguishing patients who did not benefit from ICD implantation from those who did or had a neutral outcome. The risk of non-benefit in different age groups (cut-off value derived by multivariate and ROC analysis) was assessed using the Kaplan-Meier method, and comparisons between survival curves were performed using the log-rank test. A p-value of less than 0.05 was considered statistically significant. All probability values presented are two-sided.

**Table 1:** Baseline characteristics of the study cohort (*n* = 422) divided in patients without benefit and with benefit or neutral outcome.

Variables	No Benefit (*n* = 84)	Benefit or neutral (*n* = 338)	*P* value
Age (years)	72.9 ± 6.9	65.3 ± 11.8	**< 0.001**
Women (♀), n (%)	17 (20)	69 (20)	0.971
Primary prevention, n (%)	72 (86)	251 (74)	**0.027**
Secondary prevention, n (%)	12 (14)	87 (26)	**0.027**
Early implantation (years 2011–2015), n (%)	61 (73)	178 (53)	**< 0.001**
Cardiac resynchronisation therapy, n (%)	30 (36)	104 (31)	0.384
Generator replacement, n (%)	12 (14)	72 (21)	0.150
Inadequate ICD shock, n (%)	6 (7)	24 (7)	0.989
Medical history			
Hypertension, n (%)	68 (81)	248 (73)	0.152
Diabetes mellitus, n (%)	39 (46)	109 (32)	**0.015**
Coronary artery disease, n (%)	48 (57)	193 (57)	0.949
Myocardial infarction, n (%)	32 (38)	129 (38)	0.991
Atrial fibrillation, n (%)	36 (43)	112 (33)	0.083
Stroke and/or TIA, n (%)	18 (21)	55 (16)	0.263
Chronic obstructive pulmonary disease, n (%)	20 (24)	43 (13)	**0.011**
Peripheral artery disease, n (%)	21 (25)	53 (16)	**0.044**
Kidney disease, n (%)	55 (65)	134 (40)	**< 0.001**
Anemia, n (%)	39 (46)	90 (27)	**< 0.001**
Medication			
ACEI or ARB or ARNI, n (%)	76 (90)	304 (90)	0.489
Betablocker, n (%)	78 (93)	300 (89)	0.178
Loop diuretics, n (%)	69 (82)	218 (64)	**0.001**
Aldosterone antagonist, n (%)	45 (54)	214 (63)	0.112
Amiodarone, n (%)	17 (20)	44 (13)	0.085
Echocardiography			
Left ventricular ejection fraction (%)	32 ± 9	32 ± 11	0.721
Mitral valve regugitation, n (%)	57 (68)	224 (66)	0.418
Tricuspid valve regurgitation, n (%)	42 (50)	139 (41)	0.079
Electrocardiography			
Heart rate (beats/min)	75 ± 16	76 ± 20	0.721
Sinus rhythm, n (%)	58 (69)	276 (82)	**0.021**
Left bundle branch block, n (%)	19 (23)	98 (29)	0.243
Laboratory parameters			
Hemoglobin (g/dL)	12.7 ± 1.8	13.5 ± 1.9	**< 0.001**
Creatinine (mg/dL)	1.3 ± 0.4	1.1 ± 0.4	**< 0.001**

ACEI, Angiotensin-converting-enzyme-inhibitor; ARB, Angiotensin receptor blocker; ARNI, Angiotensin-receptor-neprilysin-inhibitor; ICD, implantable cardioverter defibrillator; TIA, transient ischemic attack.

**Table 2:** Univariate analysis.

Variable	Hazard Ratio	95% Confidence Interval	*P* value
Age	1.072	1.045–1.099	**< 0.001**
Hemoglobin	0.775	0.696–0.863	**< 0.001**
Creatinine	2.411	1.557–3.734	**< 0.001**
Diabetes mellitus	1.665	1.083–2.560	**0.020**
Peripheral artery disease	2.363	1.430–3.905	**< 0.001**
Chronic obstructive pulmonary disease	2.264	1.362–3.763	**0.002**
Loop diuretics	2.028	1.140–3.606	**0.016**
Sinus rhythm	0.450	0.277–0.731	**0.001**
Anemia	2.919	1.881–4.528	**< 0.001**
Kidney disease	2.616	1.667–4.104	**< 0.001**

## Results

From 2011 to 2020, 437 patients underwent ICD implantation in the university hospital St. Josef Hospital Bochum. 15 patients (3%) were lost during follow-up and had to be excluded from the analysis due to the lack of available information. The remaining 422 patients formed the final study cohort. There was a primary prophylactic indication for ICD implantation in 323 patients (77%) and a secondary prophylactic indication in 99 patients (23%).

The mean follow-up was 4.2 ± 3.0 years. 106 patients died during follow-up (25%). Of these patients, 40 died of cardiovascular death (cardiac pump failure, *n* = 23; ventricular tachycardia/ ventricular fibrillation, *n* = 6; myocardial infarction, *n* = 4; cardiogenic sepsis, *n* = 3 [device‐associated, *n* = 1; Mitral valve endocarditis, *n* = 1; left ventricular assist device infection, *n* = 1]; vascular surgery, *n* = 2; pulmonary artery embolism, *n* = 1; other, *n* = 1). A total of 40 patients died of non-cardiovascular causes (cancer, *n* = 11; pneumonia, *n* = 9; sepsis, *n* = 7; trauma surgery, *n* = 4; suicide *n* = 1; other *n* = 8). The precise cause of death remained undetermined in 26 patients.

No benefit of ICD implantation (defined as death from any cause without prior appropriate ICD therapy) was observed in 84 patients (20%). The one-, two- and three-year no-benefit rates were 6.4%, 7.8% and 10.9%. A benefit of ICD implantation (defined as appropriate ICD therapy before death from any cause or as appropriate ICD therapy and survival to the end of the observation period) occurred in 89 patients (21%). A neutral outcome was present in 249 surviving patients (59%) without appropriate ICD therapy.

### Clinical baseline characteristics of patients with and without benefit

Out of the 422 patients included in the study, 86 were female (20%). The average left ventricular ejection fraction was 32.3 ± 10.3% (ranged from 15 to 65%). The average age of the patients was 66.9 ± 11.3 years, ranging from 22 to 89 years. At the time of implantation, 101 patients (24%) were younger than 60 years old, 118 (28%) were aged between 60 and 69 years, 158 (37%) between 70 and 79 years, and 45 (11%) were aged 80 years or older.

The group without benefit (*n* = 84, 20%) was compared to the combined group with benefit/neutral outcome (*n* = 338, 80%). Table 1 presents the clinical baseline characteristics of the patients at the time of ICD implantation. Of the 84 patients without benefit, 86% had a primary prophylactic indication for ICD implantation and only 14% had a secondary prophylactic indication. The patients with no-benefit had significantly more frequently early implantation defined as ICD implantation within the years 2011 to 2015. Patients without a benefit of ICD therapy were significantly older (72.9 ± 6.9 years vs. 65.3 ± 11.8 years, *p* < 0.001) and suffered more often from diabetes mellitus (46% vs. 32%, *p* = 0.015), chronic obstructive pulmonary disease (COPD) (24% vs. 13%, *p* = 0.011), peripheral artery disease (25% vs. 16%, *p* = 0.044), kidney disease (65% vs. 40%, *p* < 0.001), and anemia (46% vs. 27%, *p* < 0.001) than patients with benefit or neutral outcome. Consistent with the differences in comorbidities between both groups, non-benefit patients had significantly lower hemoglobin (12.7 ± 1.8 g/dL vs. 13.5 ± 1.9 g/dL, *p* < 0.001) and elevated creatinine levels (1.3 ± 0.4 mg/dL vs. 1.1 ± 0.4 mg/dL, *p* < 0.001). In addition, patients without benefit were more likely to receive loop diuretics (82% vs. 64%, *p* = 0.001). On ECG, sinus rhythm was less frequent in these patients than in patients with benefit/neutral outcome (69% vs. 82%, *p* = 0.021). Notably, no differences were observed between the groups in terms of sex, left ventricular ejection fraction or comorbidities such as coronary artery disease or myocardial infarction. The rates of CRT, generator replacement and inadequate shocks were also similar between patients without benefit and those with benefit or neutral outcome (Table 1).

### Predictors for non-benefit of ICD implantation

On univariate Cox analysis, age, hemoglobin levels, creatinine levels, diabetes mellitus, peripheral artery disease, COPD, prescription of loop diuretics, sinus rhythm, anemia, and kidney disease were significantly related to no-benefit of ICD implantation (Table 2).

Stepwise multivariate analysis identified age, anemia, peripheral artery disease, and COPD as independent risk factors of no-benefit of ICD implantation. Using ROC analysis, age cutoff value for dividing study patients were age ≥ 68 years (Area under the curve (AUC) 0.687, Confidence Interval (CI) 0.632–0.742, *p* < 0.001). The hazard ratio and confidence intervals of the independent risk factors for no-benefit of ICD implantation are given in Table 3.

**Table 3:** Multivariate analysis.

Variable	Hazard Ratio	95% Confidence Interval	*P* value
Age ≥ 68 years	4.599	2.589–8.168	**< 0.001**
Anemia	2.549	1.604–4.051	**< 0.001**
Peripheral artery disease	2.066	1.216–3.509	**0.007**
Chronic obstructive pulmonary disease	1.939	1.143–3.290	**0.014**

### Subgroup analysis – different age groups

**Fig. 1:**
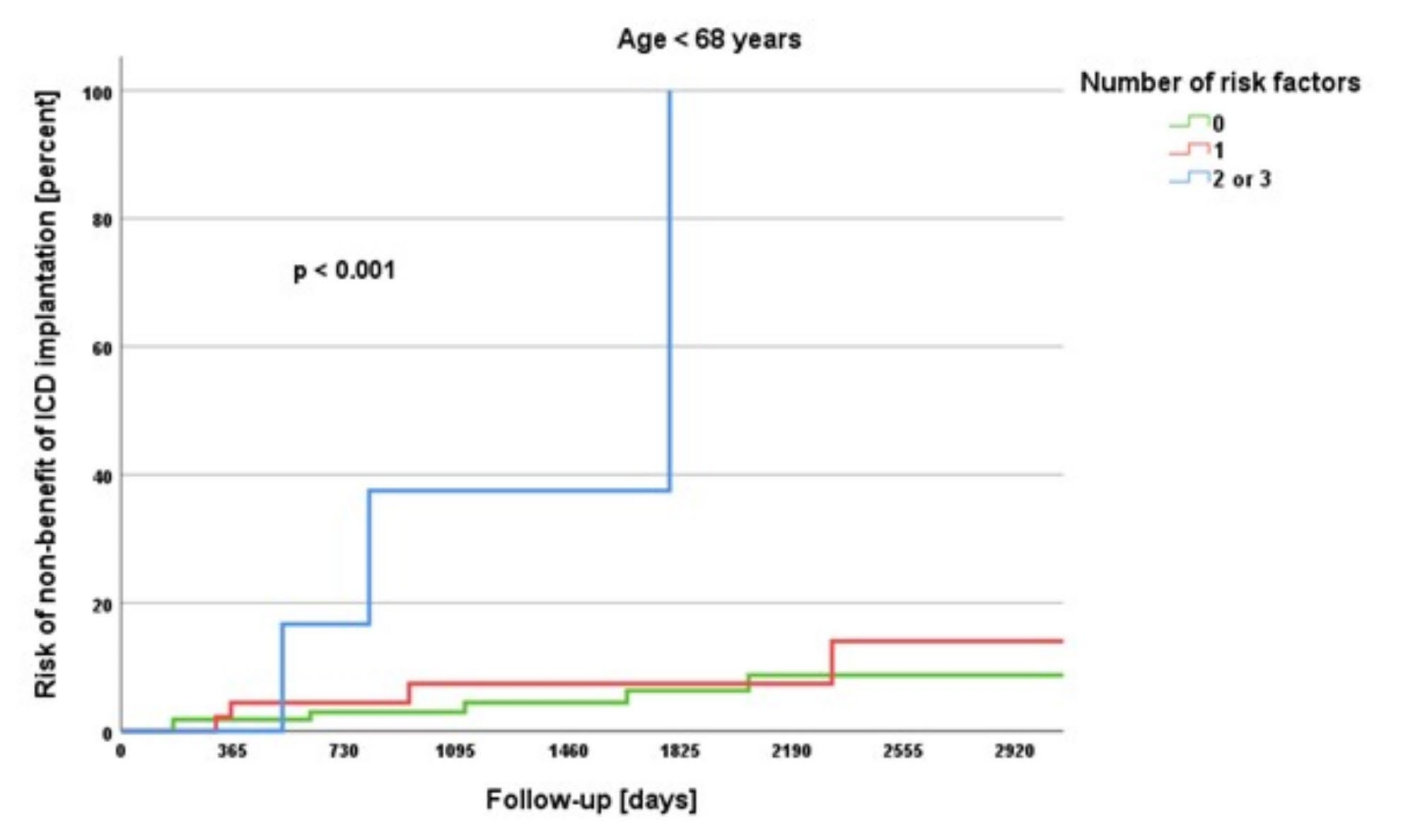
Kaplan-Meier survival analysis for age < 68 years.

**Fig. 2:**
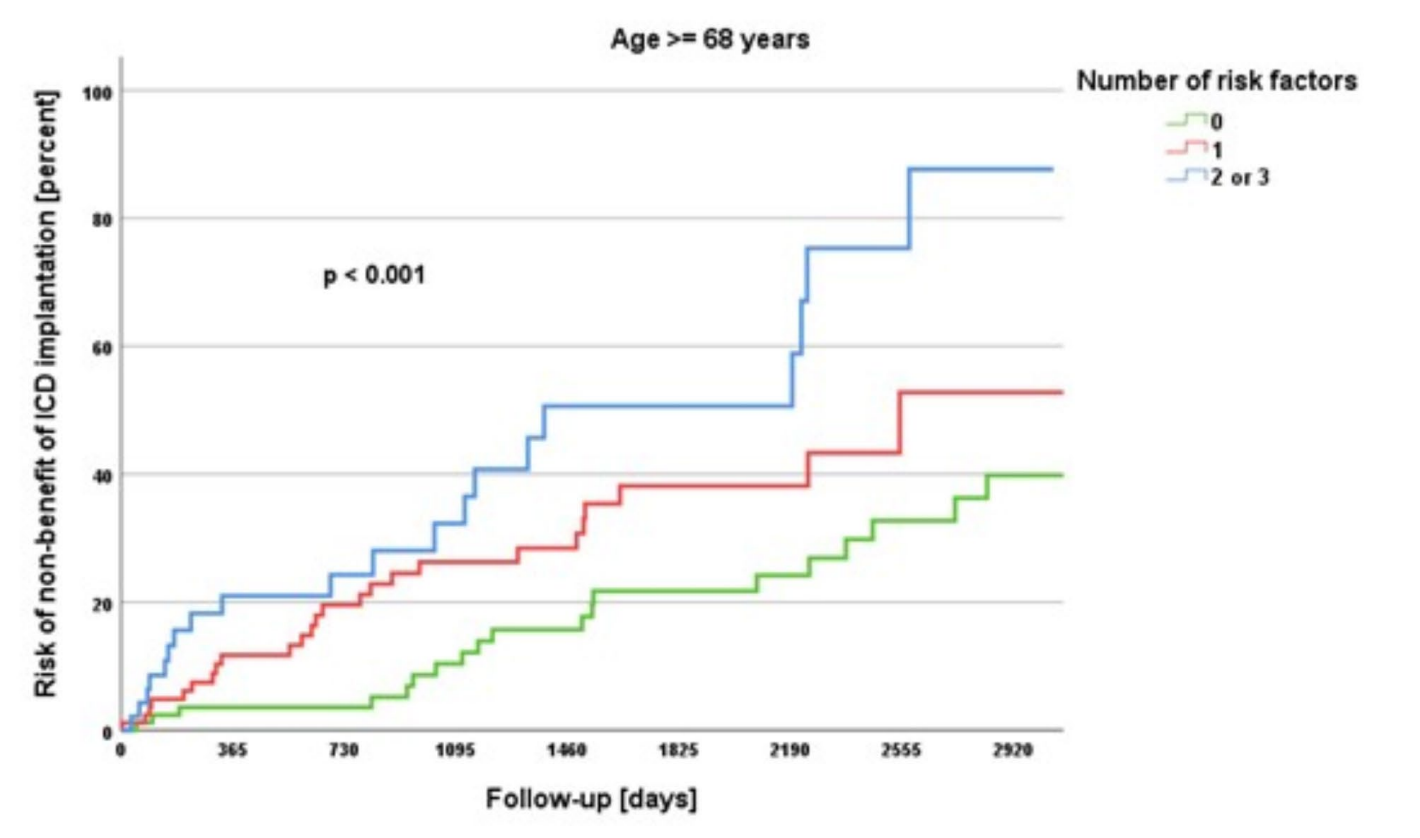
Kaplan-Meier survival analysis for age ≥ 68 years.

Kaplan-Meier survival analyses were performed for the endpoint no-benefit. Here, the study cohort was divided into patients under 68 years and 68 years and older, which was previously identified in the ROC analysis as the cut-off value for age as an independent risk factor. With increasing number of independent risk factors, the no-benefit rates increased significantly in both groups (each *p* < 0.001), however, to a greater extent in the 68 years and older group (Figs. 1 and 2). In the group under 68 years of age, the risk of non-benefit of ICD implantation was 5.6% if no risk factor was present, 7.4% if one risk factor was present and 30.0% if two or three risk factors were present (*p* < 0.001). In the 68 years and older group, the risk of non-benefit was 24.2% in the absence of any risk factor, 28.9% for one risk factor and 42.6% for two or three risk factors (*p* < 0.001).

**Fig. 3:**
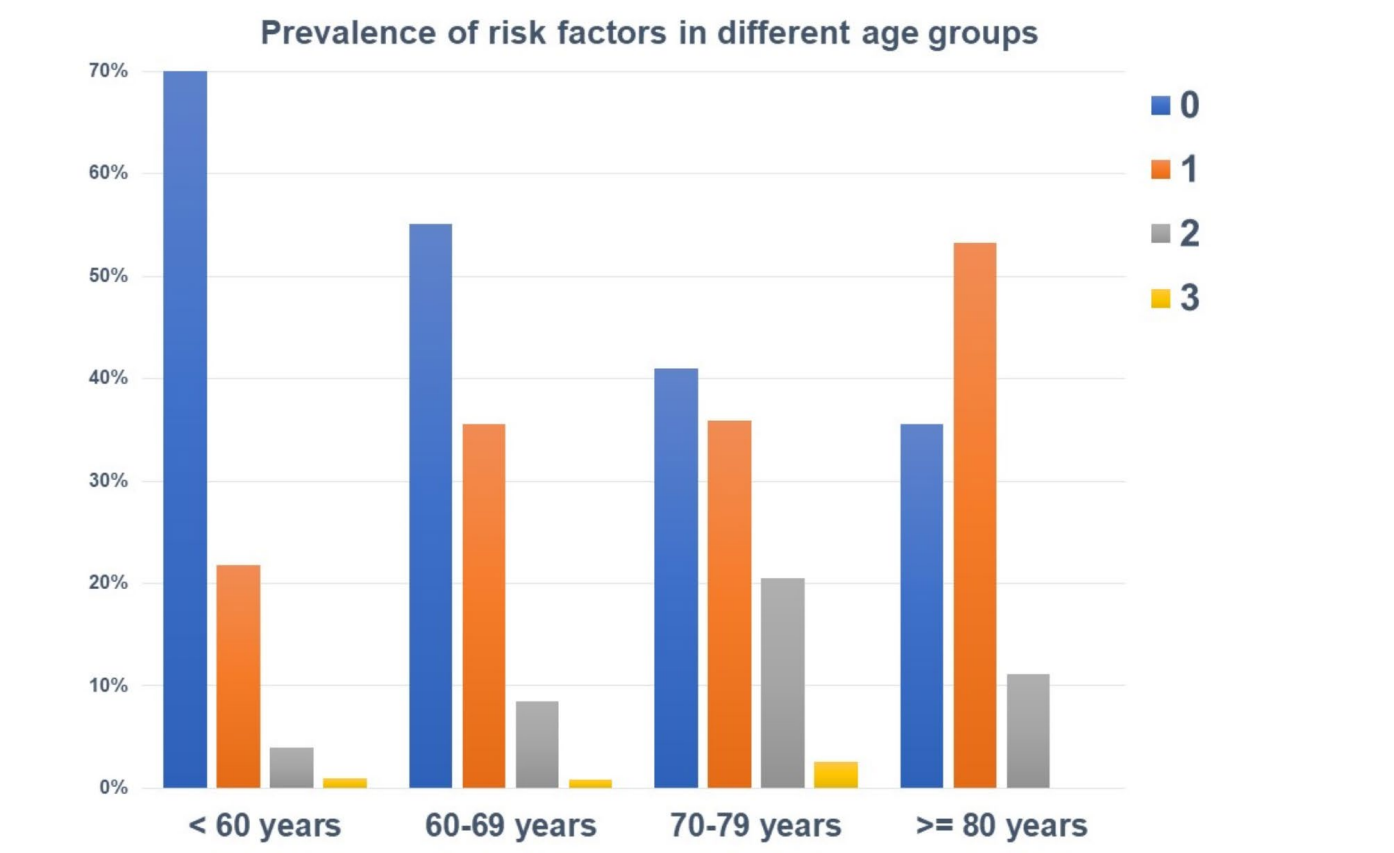
Prevalence of risk factors in different age groups.

Next, the study cohort was divided into the age groups under 60 years, 60 to 69 years, 70 to 79 years, and 80 years and older and the respective prevalence rates of the identified risk factors were illustrated, excluding age as a risk factor. With increasing age, the prevalence rates of risk factors for no-benefit of ICD implantation also increased on average. Interestingly, there was little difference between the group aged 80 years and older compared to the groups aged 70–79 years (*p* = 0.124) and 60–69 years (*p* = 0.129). However, we found higher prevalence of multiple risk factors compared to the group < 60 years (*p* < 0.001) (Fig. 3).

### Subgroup analysis – ICD implantation in 2011–2015 versus 2016–2020

The study cohort was divided into groups with early ICD implantation (years 2011–2015, *n* = 238) and late ICD implantation (years 2016–2020, *n* = 183) to detect potential trends over the decade. The mean follow-up time was 4.5 ± 3.2 years in the early implantation group and 2.3 ± 1.7 years in the late implantation group (*p* < 0.001). A total of 77 patients died in the early implantation group and 29 patients died in the late implantation group (*p* < 0.001). 61 patients (26%) in the early group and 23 patients (13%) in the late implantation group had no benefit, whereas 178 patients (74%) in the early group and 160 patients (87%) in the late group exhibited a benefit or a neutral outcome. Early implantation was significantly associated with no-benefit (Table 1), but the one-, two- and three-year no-benefit rates were 7.1%, 8.4% and 12.1% in the early implantation group compared to 5.5%, 7.1% and 9.3% in the late implantation group, which demonstrated no significant difference between the two groups (*p* = 0.493, *p* = 0.632, *p* = 0.353).

In the early implantation group, patients without benefit of ICD therapy were significantly older (72.8 ± 6.7 years vs. 65.8 ± 11.5 years, *p* < 0.001) and suffered more often from atrial fibrillation (39% vs. 26%, *p* = 0.047), peripheral artery disease (21% vs. 11%, *p* = 0.049), kidney disease (69% vs. 43%, *p* < 0.001), and anemia (43% vs. 24%, *p* = 0.004) than patients with benefit or neutral outcome. In addition, loop diuretic prescription rates were higher (82% vs. 69%, *p* = 0.037), and tricuspid valve regurgitation was more present in patients without benefit (48% vs. 40% *p* = 0.036). Consistent with the significant differences in comorbidities between the groups with and without benefit, patients without benefit were less likely to have sinus rhythm on ECG (72% vs. 84% *p* = 0.049) and had lower hemoglobin levels (13.0 ± 1.5 g/dL vs. 13.6 ± 1.7 g/dL, *p* = 0.011) and higher creatinine levels (1.3 ± 0.4 mg/dL vs. 1.2 ± 0.3 mg/dL, *p* = 0.008) at the time of implantation.

In the late implantation group, the patients without benefit of ICD implantation were also older than those with benefit or neutral outcome (72.9 ± 7.6 years vs. 65.0 ± 12.1 years, *p* = 0.003), and suffered more often from diabetes mellitus (61% vs. 28%, *p* = 0.002), COPD (30% vs. 11%, *p* = 0.008), and anemia (57% vs. 30%, *p* = 0.012). Similar to the early group, loop diuretic prescription rates were higher in patients without benefit (83% vs. 59%, *p* = 0.038), while hemoglobin levels were decreased (12.1 ± 2.2 g/dL vs. 13.3 ± 2.0 g/dL, *p* = 0.006) and creatinine levels were increased (1.3 ± 0.4 mg/dL vs. 1.1 ± 0.4 mg/dL, *p* = 0.018). Notably, 21 of the 23 patients (91%) without a benefit had a primary prophylactic indication for ICD implantation and only 2 patients (9%) a secondary prophylactic indication (Table 4).

**Table 4:** Clinical characteristics of study patients (*n* = 422) divided in early (2011–2015, *n* = 239) and late implantation (2016–2020, *n* = 183).

Variable	**Early implantation (n = 239)**			Late implantation (n = 183)		
	No Benefit (n = 61)	Benefit or neutral (n = 178)	*P* value	No Benefit (n = 23)	Benefit or neutral (n = 160)	*P* value
Age (years)	72.8 ± 6.7	65.8 ± 11.5	**< 0.001**	72.9 ± 7.6	65.0 ± 12.1	**0.003**
Women (♀), n (%)	15 (25)	39 (22)	0.666	2 (9)	30 (19)	0.378
Primary prevention, n (%)	51 (84)	141 (79)	0.456	21 (91)	110 (69)	**0.026**
Secondary prevention, n (%)	10 (16)	37 (21)	0.456	2 (9)	50 (31)	**0.026**
Cardiac resynchronisation therapy, n (%)	20 (33)	52 (29)	0.600	10 (43)	52 (33)	0.298
Inadequate ICD shock, n (%)	4 (7)	15 (8)	0.788	2 (9)	9 (6)	0.632
Medical history						
Hypertension, n (%)	47 (77)	130 (73)	0.537	21 (91)	118 (74)	0.072
Diabetes mellitus, n (%)	25 (41)	64 (36)	0.483	14 (61)	45 (28)	**0.002**
Coronary artery disease, n (%)	37 (61)	106 (60)	0.953	11 (48)	87 (54)	0.700
Myocardial infarction, n (%)	22 (36)	72 (40)	0.545	10 (43)	57 (36)	0.465
Atrial fibrillation, n (%)	24 (39)	47 (26)	**0.047**	12 (52)	65 (41)	0.306
Stroke and/or TIA, n (%)	12 (20)	22 (12)	0.158	6 (26)	33 (21)	0.550
Chronic obstructive pulmonary disease, n (%)	13 (21)	26 (15)	0.221	7 (30)	17 (11)	**0.008**
Peripheral artery disease, n (%)	13 (21)	20 (11)	**0.049**	8 (35)	33 (21)	0.128
Kidney disease, n (%)	42 (69)	77 (43)	**< 0.001**	13 (57)	57 (36)	0.054
Anemia, n (%)	26 (43)	42 (24)	**0.004**	13 (57)	48 (30)	**0.012**
Medication						
ACEI or ARB or ARNI, n (%)	55 (90)	159 (89)	0.607	21 (91)	145 (91)	1.000
Betablocker, n (%)	56 (92)	165 (93)	1.000	22 (96)	135 (84)	0.207
Loop diuretics, n (%)	50 (82)	123 (69)	**0.037**	19 (83)	95 (59)	**0.038**
Aldosterone antagonist, n (%)	33 (54)	111 (62)	0.290	12 (52)	103 (64)	0.241
Amiodarone, n (%)	15 (25)	26 (15)	0.068	2 (9)	18 (11)	1.000
Echocardiography						
Left ventricular ejection fraction (%)	31 ± 9	32 ± 10	0.673	34 ± 8	33 ± 11	0.737
Mitral valve regugitation, n (%)	40 (66)	115 (65)	0.637	17 (74)	109 (68)	0.751
Tricuspid valve regurgitation, n (%)	29 (48)	71 (40)	**0.036**	13 (57)	68 (43)	0.295
Electrocardiography						
Heart rate (beats/min)	75 ± 17	76 ± 21	0.771	74 ± 13	76 ± 20	0.825
Sinus rhythm, n (%)	44 (72)	150 (84)	**0.049**	14 (61)	126 (79)	0.089
Left bundle branch block, n (%)	14 (23)	59 (33)	0.136	5 (22)	39 (24)	0.782
Laboratory parameters						
Hemoglobin (g/dL)	13.0 ± 1.5	13.6 ± 1.7	**0.011**	12.1 ± 2.2	13.3 ± 2.0	**0.006**
Creatinine (mg/dL)	1.3 ± 0.4	1.2 ± 0.3	**0.008**	1.3 ± 0.4	1.1 ± 0.4	**0.018**

ACEI, Angiotensin-converting-enzyme-inhibitor; ARB, Angiotensin receptor blocker; ARNI, Angiotensin-receptor-neprilysin-inhibitor; ICD, implantable cardioverter defibrillator; TIA, transient ischemic attack.

Significant values are in bold.

Multivariate analyses identified age ≥ 70 years (HR 2.756, 95% CI 1.573–4.830, *p* < 0.001), atrial fibrillation (HR 2.278, 95% CI 1.315–3.945, *p* = 0.003), peripheral artery disease (HR 2.263, 95% CI 1.155–4.432, *p* = 0.017), and anemia (HR 2.998, 95% CI 1.730–5.194, *p* < 0.001) as independent risk factors for non-benefit of ICD implantation in the early group, and age ≥ 66 years (HR 5.936, 95% CI 1.763–19.985, *p* = 0.004), diabetes mellitus (HR 4.362, 95% CI 1.756–10.831, *p* = 0.002), COPD (HR 4.194, 95% CI 1.651–10.655, *p* = 0.003), and anemia (HR 2.482, 95% CI 1.060–5.814, *p* = 0.036) as independent risk factors for non-benefit of ICD implantation in the late group (Tables 5, 6, 7 and 8, Supplemental Figs. 1–4).

**Table 5:** Univariate analysis for early implantation.

Variable	Hazard Ratio	95% Confidence Interval	*P* value
Age	1.072	1.040–1.105	< 0.001
Atrial fibrillation	2.575	1.519–4.363	< 0.001
Peripheral artery disease	2.473	1.329–4.601	0.004
Sinus rhythm	0.457	0.254–0.823	0.009
Hemoglobin	0.749	0.647–0.866	< 0.001
Creatinine	2.334	1.349–4.039	0.002
Kidney disease	2.731	1.586–4.702	< 0.001
Anemia	2.918	1.734–4.909	< 0.001

**Table 6:** Mutivariate analysis for early implantation.

Variable	Hazard Ratio	95% Confidence Interval	*P* value
Age ≥ 70 years	2.756	1.573–4.830	< 0.001
Atrial fibrillation	2.278	1.315–3.945	0.003
Peripheral artery disease	2.263	1.155–4.432	0.017
Anemia	2.998	1.730–5.194	< 0.001

**Table 7:** Univariate analysis for late implantation.

Variable	Hazard Ratio	95% Confidence Interval	*P* value
Age	1.070	1.023–1.119	0.003
Diabetes mellitus	4.149	1.710–10.067	0.002
Chronic obstructive pulmonary disease	3.753	1.531–9.205	0.004
Hemoglobin	0.806	0.681–0.955	0.013
Creatinine	2.745	1.296–5.817	0.008
Kidney disease	2.521	1.104–5.757	0.028
Anemia	2.904	1.272–6.629	0.011

**Table 8:** Multivariate analysis for late implantation.

Variable	Hazard Ratio	95% Confidence Interval	*P* value
Age ≥ 66 years	5.936	1.763–19.985	0.004
Diabetes mellitus	4.362	1.756–10.831	0.002
Chronic obstructive pulmonary disease	4.194	1.651–10.655	0.003
Anemia	2.482	1.060–5.814	0.036

## Discussion

The present study investigated 422 patients who underwent ICD implantation from 2011 to 2020. The main findings were that (1) a considerable portion of patients (20%) experienced no benefit from ICD implantation, with significantly higher rates in the early implantation group (2011–2015), (2) patients without benefit from ICD therapy were significantly older and had a higher prevalence of comorbidities, and (3) independent risk factors for no benefit from ICD implantation were age ≥ 68 years, anemia, peripheral artery disease, and COPD. Moreover, subgroup analysis by age showed increasing risk of no benefit with age and comorbidities. Comparing patients who received ICD implantation in 2011–2015 with those in 2016–2020, there were no significant differences in the rates of no benefit over one-, two-, and three-year periods. However, certain comorbidities were associated with no benefit in both early and late implantation groups. In the early implantation group, age ≥ 70 years, atrial fibrillation, peripheral artery disease, and anemia were independent risk factors for no benefit, while in the late implantation group, age ≥ 66 years, diabetes mellitus, COPD, and anemia were identified as independent risk factors.

Our analysis provides several important aspects. First, it covers a trend that extends over an entire decade, providing valuable insights into the development of ICD patient collectives and selection over time. Second, we focused on a detailed analysis of comorbidities, which has been less frequently considered in previous studies. Third, our study comprises different subgroups, in terms of age and time of implantation, which allows a differentiated analysis of therapeutic response in different patient populations. In addition, our study provides an extended inclusion period and a long follow-up period, even for patients in the late-implant group.

We included all patients who had an implantable cardioverter defibrillator (ICD) implanted in our hospital between 2011 and 2020. Remarkably, only 3% of patients were lost to follow-up, thus providing a robust data set for analysis.

Therefore, our study offers valuable insights into a cohort of patients implanted with an ICD according to standard clinical recommendations. By examining trends over a decade, we can better assess the long-term effectiveness or ineffectiveness of ICD therapy and identify changes in the composition of ICD patient populations in a real-world clinical setting.

In the present study, the no-benefit rate was 20%. This rate may be slightly lower, although comparable with other studies that demonstrated rates between 23 and 28%^15,16^.

In this analysis, age ≥ 68 years, anemia, peripheral artery disease, and COPD were identified as independent risk factors of no-benefit of ICD implantation. Previous studies have already indicated that the presence and number of comorbidities play a major role in the prognosis of ICD patients, both in terms of overall mortality and benefit from ICD therapy^16,17^. On the one hand, comorbidities and even high comorbidity burden have not necessarily been shown to correlate with increased rates of appropriate ICD therapy, but on the other hand, all-cause mortality rates, rates of no benefit, and rates of inappropriate ICD therapy also increase with increasing comorbidity^11,16–20^. In addition, it is known that very old patients are less likely to benefit from ICD therapy due to limited remaining lifespan and increased likelihood of non-arrhythmogenic death because of competing risk factors^18,21^.

An analysis from the Israeli ICD Registry involving 2617 patients revealed several independent risk factors for one-year mortality, including age greater than 75 years, atrial fibrillation, chronic lung disease, anemia, and chronic renal failure^22^. In another study, the researchers sought to assess the outcomes following ICD generator replacement^23^. Among the 1421 patients undergoing ICD generator replacement (with a mean age of 69.6 years and 81% male), appropriate therapy occurred after replacement in 435 patients (30.6%) over an average follow-up period of 2.7 years^23^. Factors associated with the occurrence of appropriate therapy included a lower left ventricular ejection fraction and a history of appropriate therapy before generator replacement^23^. However, death before receiving appropriate ICD therapy was observed in 336 patients (23.7%)^23^. Factors contributing to a higher risk of non-benefit included older age, lower left ventricular ejection fraction, and noncardiac comorbidities such as diabetes mellitus, chronic lung disease, peripheral vascular disease, lower hemoglobin levels, and decreased glomerular filtration rate^23^. The study noted a progressive increase in mortality with an accumulation of these noncardiac comorbidities^23^. In the present study, almost identical risk factors for non-benefit of ICD therapy were identified. We were able to confirm the results, albeit over a much longer follow-up period, which increased the robustness of the identified risk factors for non-benefit. To sum up, multimorbidity can significantly reduce the effectiveness of ICD therapy. Patients with multiple comorbidities often have a reduced life expectancy, implying that they may not live long enough to benefit from ICD therapy. In addition, many of these comorbidities, such as diabetes, kidney disease and COPD, are non-cardiac conditions and may contribute to a higher risk of noncardiac mortality, reducing the potential benefit of ICD therapy.

In recent years, the proportion of ICD implantations among patients over 80 years old increased^24,25^. However, age represents a risk factor for non-benefit of ICD therapy, as there are more competing, life-limiting risk factors in older age^21^. Therefore, it is difficult to determine a cut-off value based on age alone when an elderly patient is no longer likely to benefit from ICD therapy. In our study, this is demonstrated by a moderate AUC value for the age cut-off of 68 years for non-benefit.

The DANISH trial aimed to investigate the benefit of prophylactic ICD in patients with symptomatic systolic heart failure not attributable to coronary artery disease^9^. The study included 556 patients who received an ICD and 560 patients who received usual clinical care, with 58% in both groups receiving CRT^9^. After a median follow-up of 67.6 months, the trial found that prophylactic ICD implantation did not lead to a significantly lower rate of death from any cause compared to usual clinical care (HR, 0.87; *p*= 0.28)^9^. However, there was a significant reduction in sudden cardiac death in the ICD group compared to the control group (HR, 0.50; *p*= 0.005)^9^. Notably, among patients younger than 68 years old, the mortality rate from all causes was notably lower in the ICD group compared to the control group (HR, 0.64; *p*= 0.01)^9^. In the present study, we also identified 68 years as the cut-off value for distinguishing between no-benefit and benefit/neutral outcomes of ICD therapy. We subsequently evaluated the prevalence of the identified risk factors for non-benefit across different age groups and conducted Kaplan-Meier survival analyses for age groups younger than 68 years and 68 years and older. We observed that the number of risk factors increased with age, with little variation between the groups over 60 years, over 70 years and over 80 years. Additionally, it became evident that the number of risk factors had a significant impact on the benefit of ICD therapy, both in the group younger than 68 years and in the group aged 68 years and older, with the effects being even more pronounced in the older group. However, the results also highlight that a patient under 68 years old with two or three risk factors may have a similar or even greater likelihood of non-benefit from ICD therapy compared to a patient over 68 years old with no or an additional risk factor besides age. Advanced age alone should not be the sole criterion for withholding ICD implantation, so 80-year-olds should not be necessarily denied an ICD^21^. The findings imply that around the age of 70 years, a more thorough evaluation of ICD implantation is warranted.

In this study, the lower implantation rate in the late implantation group may be influenced by randomized-controlled ICD studies in the mid-last decade like the DANISH trial which demonstrated that prophylactic ICD implantation did not lead to a significantly lower rate of death from any cause^9^. Comorbidities are becoming increasingly important for patient selection, leading to more restrictive indication criteria. There were fewer comorbidities associated with non-benefit in the late group compared to earlier years, suggesting a potential improvement in patient selection.

In addition, the early implantation group had significantly higher rates of no benefit compared to the late implantation group. This association could be caused by the different follow-up periods, as the one-, two- and three-year rates without benefit did not differ significantly between the two groups.

## Limitations

An important limitation of the study is that it is a monocentric analysis, which could potentially have resulted in a selection bias in patient undergoing ICD implantation. Unfortunately, no established frailty indices or comorbidity indices could be applied in this analysis, due to the retrospective nature of the study and the fact that not all factors included in the indices were recorded. In these established scores, dementia and tumor diseases are significant comorbidities. However, these comorbidities may also be contraindications for implantation of an ICD, so that we did not include patients with these comorbidities. Another important limitation is the relatively small sample size, particularly in patients older than 80 years. In part, this can be attributed to the more restrictive approach to patients of advanced age - an approach that is supported by the results of the present study, especially in the presence of further risk factors. In general, it should also be considered that an ICD shock alone is not a measure of the success of ICD therapy. The benefit of ICD therapy can only be properly assessed by comparing the survival of patients with and without an ICD.

## Conclusion

In summary, this study highlights that while ICD therapy can be beneficial, a significant proportion of patients may not derive the expected benefit. Risk factors such as older age and certain comorbidities are associated with a higher likelihood of no-benefit from ICD implantation. Our study emphasizes the importance of age for the potential non-benefit of ICD therapy. However, even young patients with a high burden of comorbidities have a high risk of not benefiting from ICD therapy. This trend has not changed in the last 10 years despite the more cautious indication for ICD implantation.

Further research is needed to refine patient selection criteria and ensure the effectiveness of therapy while minimizing potential risks and healthcare costs for those who are not likely to benefit from ICD therapy.

## Electronic supplementary material

Below is the link to the electronic supplementary material.

Supplementary Material 1

## Data Availability

The data that support the findings of this study are not openly available due to reasons of sensitivity and are available from the corresponding author upon reasonable request.

## Abbreviations

ATP: Antitachycardia pacing
CRT: Cardiac resynchronization therapy
ECG: Electrocardiogram
eGFR: Estimated glomerular filtration rate
ICD: Implantable cardioverter defibrillator
