# Supplementary material for: Risk factors for non-benefit of implantable cardioverter defibrillator therapy
Source: Sci Rep. 2025 Jan 20;15:2480. doi: 10.1038/s41598-025-86022-x (PMC11756404; doi:10.1038/s41598-025-86022-x)

**Supplemental figure 1:** Kaplan-Meier survival analysis for age < 70 years in patients with early ICD implantation


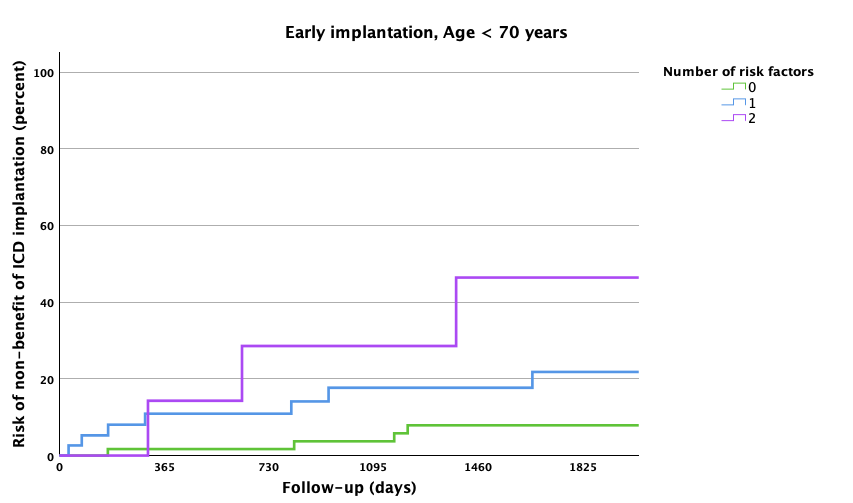


**Supplemental figure 2:** Kaplan-Meier survival analysis for age >= 70 years in patients with early ICD implantation


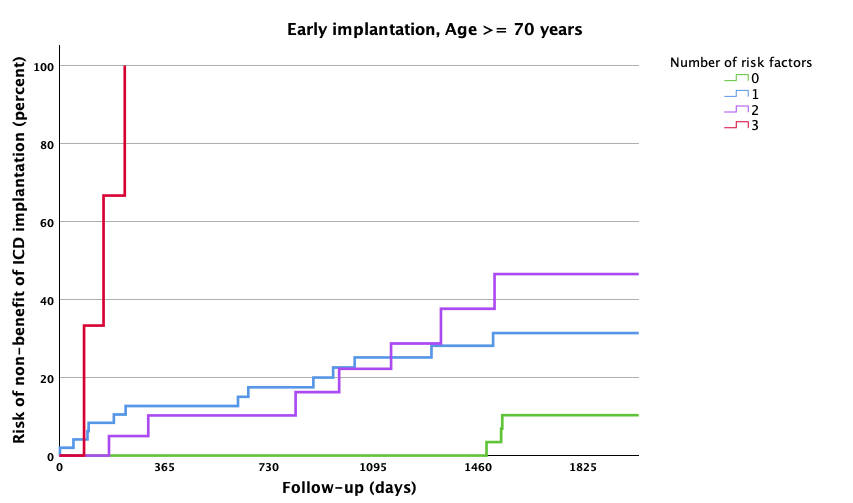


**Supplemental figure 3:** Kaplan-Meier survival analysis for age < 66 years in patients with late ICD implantation


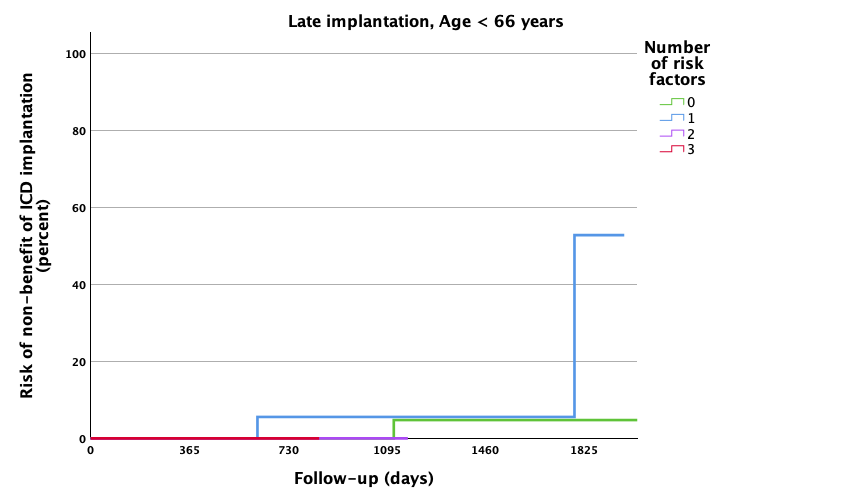


**Supplemental figure 4:** Kaplan-Meier survival analysis for age >= 66 years in patients with late ICD implantation


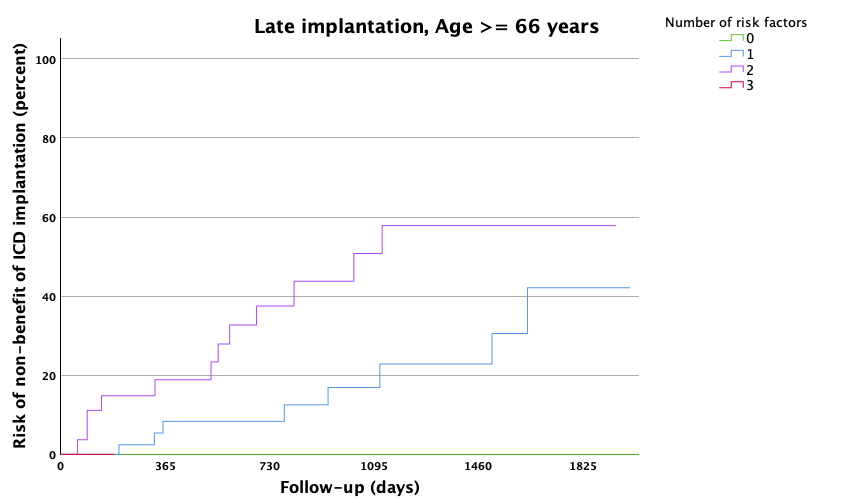

Supplement: Supplementary file 1 — Supplementary Material 1 [file 41598_2025_86022_MOESM1_ESM.docx]
